# ALLTogether recommendations for biobanking samples from patients with acute lymphoblastic leukaemia: a modified Delphi study

**DOI:** 10.1038/s41416-025-02958-x

**Published:** 2025-02-22

**Authors:** Amélie Trinquand, James Leveson, Ana Lúcia Barbosa, Paula Gameiro, Tiina Vesterinen, Tim Lammens, Thomas Drost, Anthony V. Moorman, Valérie de Haas, Jonathan Bond, Judith M. Boer, on behalf of the Biobank Committee of the ALLTogether Consortium, Amélie Trinquand, Ana Lúcia Barbosa, Paula Gameiro, Tiina Vesterinen, Tim Lammens, Anthony V. Moorman, Jonathan Bond, Judith M. Boer, Valérie de Haas, Marion Persoon, Anne Thomson, Karolin Bergenstråhle, Maria Thastrup, Udo zur Stadt, Lena Behrmann, Anne Uyttebroeck, Aurélie Caye, Marion Strullu, Cristina Jou Munoz, Anna Castleton

**Affiliations:** 1 National Children’s Cancer Service, Children’s Health Ireland at Crumlin, Dublin, Ireland; 2 University College London NHS Trust, London, UK; 3 Department of Haematology, Instituto Português de Oncologia, Lisbon, Portugal; 4 Institute for Molecular Medicine Finland (FIMM), HiLIFE, University of Helsinki, Helsinki, Finland; 5 Department of Internal Medicine and Pediatrics, Ghent University, 9000 Ghent, Belgium; 6 Department of Pediatric Hematology-Oncology and Stem Cell Transplantation, Ghent University Hospital, 9000 Ghent, Belgium; 7 Cancer Research Institute Ghent, 9000 Ghent, Belgium; 8 ALLTogether Patient and Public Involvement in Research (PPI) representative, Utrecht, The Netherlands; 9 Leukaemia Research Cytogenetics Group, Centre for Cancer, Translational and Clinical Research Institute, Newcastle University, Newcastle upon Tyne, UK; 10 Princess Máxima Center for Pediatric Oncology, Utrecht, The Netherlands; 11 Systems Biology Ireland, School of Medicine, University College Dublin, Dublin, Ireland; 12 VIVO Biobank, Milton Keynes, UK; 13 Uppsala Biobank, Uppsala University, Uppsala, Sweden; 14 Department of Pediatrics and Adolescent Medicine, Rigshospitalet, University of Copenhagen, Copenhagen, Denmark; 15 Paediatric Haematology and Oncology, University Medical Centre Hamburg-Eppendorf, Hamburg, Germany; 16 Department of Paediatric Haematology and Oncology, University Hospitals Leuven, Leuven, Belgium; 17 Service de de Génétique Moléculaire, Hôpital Robert Debré, GHU AP-HP Nord - Université Paris Cité, Paris APHP-Centre de ressources biologiques (CRB), Hôpital Robert Debré, Paris, France; 18 Service d’Hémato-Immunologie pédiatrique, Hôpital Robert Debré, GHU AP-HP Nord - Université Paris Cité, Paris, France; 19 Hospital Niño Jesús, Madrid, Spain; 20 Haematology Department, The Christie Hospital NHS Foundation Trust, Manchester, UK

**Keywords:** Haematological cancer, Translational research

## Abstract

Acute lymphoblastic leukaemia (ALL) is a rare and heterogeneous disease. The ALLTogether consortium has implemented a treatment protocol to improve outcome and reduce treatment-related toxicity across much of Europe. The consortium provides the opportunity to design translational research on patient material stored in national biobanks. However, there are currently no standardized guidelines for the types of material, processing, and storage for leukaemia biobanking. To address this gap, we conducted a modified Delphi survey among 53 experts in different roles related to leukaemia. The first round consisted of 63 statements asking for level of agreement. The second round refined some to reach consensus, using yes-no and multiple-option answers. Key recommendations include cryopreservation of cells from diagnosis, post-induction, post-consolidation, and relapse, with at least two aliquots of plasma and serum, and cerebrospinal fluid from diagnosis, day15, and post-induction. It was advised to distribute cells across multiple vials for various research projects, and to collect data on sample processing, cell viability, and blast percentage. Quality monitoring and user feedback were strongly recommended. The Delphi survey resulted in strong recommendations that can be used by national biobanks to harmonize storage of samples from patients with ALL and ensure high-quality cryopreserved cells for research studies.

## Introduction

The ALLTogether consortium has implemented a standard treatment protocol, ALLTogether1, for acute lymphoblastic leukaemia (ALL) in children, adolescents, and young adults across Northern and Western Europe (NCT03911128), expecting to enrol around 6430 patients over 5 years’ time period. Establishment of this collaborative consortium has opened avenues for a multitude of scientific collaborations, particularly through the biobanking of samples from patients enrolled in the clinical trial. Biobanks play a crucial role in cancer research and personalized medicine, emphasized by The International Agency for Research on Cancer (IARC) stating that biobanks serve as a cornerstone for advancement of three expanding areas within biomedical science. These include: (i) molecular and genetic epidemiology (focusing on investigating the interplay between genetic and environmental factors in cancer causation among both the general population and familial contexts), (ii) molecular pathology (aiming to develop molecular-driven methods for classifying and diagnosing various cancers) and (iii) pharmacogenomics/pharmacoproteomics which seeks to elucidate the relationship between an individual patient's genetic makeup or observable characteristics and their response to drug therapies [1].

To facilitate ALLTogether scientific research projects, the consortium has strongly recommended that at least 50% of samples from ALLTogether1 study patients who consented for storage of biomaterial, should be reserved for collaborative ALLTogether studies, ensuring equitable access to biobanked resources across the consortium and the opportunity to study rare ALL subtypes in the context of a uniformly treated patient population. The ALLTogether1 protocol contains several sub-studies which require the biobanking of various samples -leukaemic and non-leukaemic- from peripheral blood (PB), bone marrow (BM) and cerebrospinal fluid (CSF) at critical time-points: diagnosis, day 15, post-induction therapy (day 29), post-consolidation therapy (day 71) and relapse. The ALLTogether Scientific Committee is in charge of the evaluation of collaborative research proposals requiring ALLTogether biobanked samples. As ALLTogether research projects inherently involve more than one regional study group of the consortium leading to requesting samples from several national biobanks, developing guidelines seemed crucial. To address this, the ALLTogether consortium established a Biobank Committee, comprising national representatives from each country participating in the trial. The mission of the Committee is to outline recommended practices in biobanking materials from ALL patients with the aim to enhance the quality of stored materials for research purposes, promote uniformity to facilitate joint analysis of rare subtypes, and contribute to improved patient treatment through research enabled by the biobanks. Current recommendations in oncology are not-disease specific [1]. The absence of specific recommendations for leukaemia further emphasizes the necessity to develop recommendations for biobanking across the consortium.

To achieve this objective, the ALLTogether Biobank Committee has undertaken a modified Delphi study, involving a panel of national experts from a range of disciplines. The purpose of the survey was to establish the best practice for all aspects of biobanking for ALL, free from constraints such as funds or storage space.

## Methods

### Writing group and expert panel

The ALLTogether Biobank Committee comprises representatives from countries or study groups participating in the clinical trial (Belgian Society of Paediatric Haematology Oncology (BSPHO), Nordic Society of Paediatric Haematology and Oncology (NOPHO), Société Française de lutte contre les Cancers et leucémies de l’Enfant et de l’adolescent (SFCE), Co-operative study group for childhood acute lymphoblastic leukemia (CoALL), The Paediatric Haematology/Oncology Association of Ireland (PHOAI), Princess Máxima Center for Pediatric Oncology (PMC), Grupo Português de Leucemias Pediatricas member of the Sociedade de Hematologica e Oncologia Pediatrica (GPLP-SHOP), Sociedad Española de Hematología y Oncología Pediátricas (SEHOP) and The United Kingdom ALL Group (UKALL)). Within the Committee, a dedicated writing group was tasked with developing questions for the Delphi survey. This group comprised 9 members from 6 countries (Belgium, Finland, Ireland, Portugal, The Netherlands, and The United Kingdom) with diverse roles including clinicians, researchers, laboratory scientists, and biobank coordinators. The panel of experts (*n* = 53) consisted of national experts (3 from Portugal; 4 from Germany and Ireland; 5 from Belgium, Finland, Spain and Sweden, 6 from The Netherlands; and 8 from France and The United Kingdom), nominated by their ALLTogether consortium regional representatives, including members of the Biobank Committee who did not participate in drafting the surveys. National experts represented biobank technical staff as well as users from a range of disciplines, including laboratory technicians, biobank coordinators, clinicians and biomedical scientists.

### Survey design

**Fig. 1:**
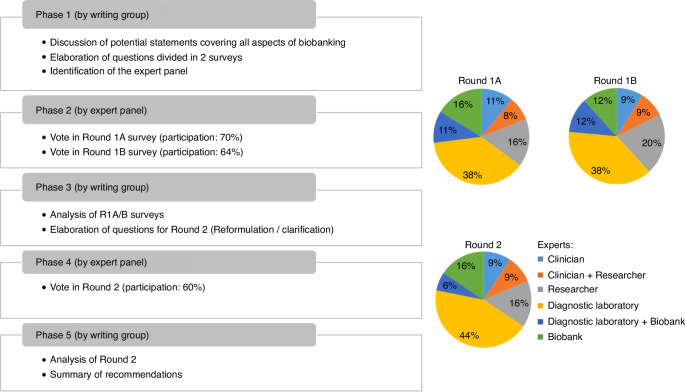
Flow chart of the Delphi process and characteristics of voting expert panel for the 2 Delphi rounds. The left panel describes the 5 phases of the Delphi process. Pies charts describe the range of disciplines of experts who responded to Round 1A, 1B and 2.

To derive consensus-based recommendations, a modified version of the Delphi method [2–4] was employed. The modification consisted of predefined items and options elaborated by the writing group instead of an open-ended questionnaire. The voting process by the expert group was facilitated by the digital survey and reporting platform Webropol. The study encompassed two Delphi rounds (Fig. 1). Round 1, split over questionnaires 1A and 1B, comprehensively covered all aspects of biobanking. Round 1A of the survey comprised questions relating to BM aspiration procedure, sampling, viable cell storage, and DNA and RNA storage. Round 1B questions covered plasma, serum, CSF, germline material, sample processing data and quality monitoring. Round 2 revisited and refined some previous questions, to clarify issues arising during the first voting rounds, and to arrive at consensus. Participants were asked by email to participate through a web link to the survey or QR code and urged to only answer to questions that were relevant to their expertise. The voting was anonymous, independent per survey, and answers were only visible by moderators of the survey (AT and JL). The anonymized answers were available to all writing group members (Supplementary Table 1).

### Consensus scoring

For Rounds 1A and 1B, the level of Agreement (LoA) score was assessed on a Likert scale of 1 (completely disagree) to 5 (completely agree). Participants had the option to abstain from answering if they lacked particular opinion or expertise. The abstention rate was calculated per recommendation by summarizing the number of participants with no opinion on the recommendation divided by the number of people who answered the question. LoA score was calculated by dividing the number of participant answering agree/strongly agree (4/5) or disagree/strongly disagree (1/2) with the total number of the participants answering the recommendation. Consensus was considered achieved when ≥66% of the participants either agreed/strongly agreed (4/5) or disagreed/strongly disagreed (1/2). For Round 2, direct questions were posed to seek consensus with options including Yes/No/No opinion as well as multiple-choice questions to gain further insight into preferred methods. Participants were encouraged to provide comments alongside with their responses, as this serves as a valuable source of information and advice. The questions and answers per participant of the three surveys are presented in Supplementary Table 1.

## Results

### Biobanking infrastructures

The Biobank Committee initially gathered information on national or study group biobanks within the consortium, focusing on their structures, stored sample types, sample processing protocols, and sample processing data. This revealed a diverse range of structures among the 10 countries involved in 9 study groups (Finland and Sweden are part of the NOPHO study group) (Supplementary Table 2). Most biobanks are centralized within countries (7/10) while three countries comprise multiple biobank locations. In countries with multiple biobanks, these biobanks are attached to a diagnostic laboratory. Central biobanks are either affiliated with a diagnostic laboratory (3/7) or operate as a separate entity (4/7). The types of stored material vary across countries. The only commonality identified in the current practice is the storage of viable frozen cells that can be used for functional analyses and for DNA, RNA and protein extraction.

### Delphi survey

The panel was asked to answer questions in 7 categories related to biobanking of ALL samples. The participation rate of the three successive surveys was 70% (37/53), 64% (34/53) and 60% (32/53) with similar distribution of panel member expertise (Fig. 1). Among all items of Round 1A and 1B, 48 out of 63 (75%) reached a positive consensus, while 15 out of 63 (24%) did not reach consensus. Subsequently, the writing group reformulated the latter questions and developed 24 new questions (Round 2) to enhance clarity and obtain additional information on reasons for the non-consensus. To this end, questions with multiple options alongside with the possibility to add comments were implemented. The revised version obtained an affirmative consensus for 18 items, while 6 items still did not reach consensus. The result for each item is summarized in Table 1 and discussed below per category, highlighting additional comments provided by the panel. The geographical spread of responders is illustrated in Supplementary Figure.

**Table 1:** Statements and results of the Delphi consensus process.

Statements	Level of agreement (%)	Rounds	Abstention rate %
1. Sample procedure and tube collection			
1.1. Bone marrow Procedure: If large sample volumes are required (e.g., for multiple diagnostic tests), it is necessary to change the bone marrow aspiration site to avoid haemodilution	95% (21/22)	R2	31% (10/32)
1.2. The needle should be reinserted at different angles through the original puncture site	82% (14/17)	R2	19% (4/21)
1.3. For storage of viable cells, the preferred tube is: Sodium Heparin or EDTA or Acid Citrate Dextrose (ACD)	No consensus	R1A and R2	22% (7/32)
1.4. The material should be taken in Coagulation/serum tubes or clot activator tubes for serum	No consensus	R1A and R2	48% (15/32)
1.5. The type of collection tube should be noted	91% (30/33)	R1A	11% (4/37)
1.6. Time between sampling and processing should be noted	81% (29/36)	R1A	3% (1/37)
1.7. Storage and transport temperature till processing should be noted	70% (23/33)	R1A	11% (4/37)
2. Biobanking of cells, DNA and RNA			
2.1. Biobanking of cells from preferably bone marrow (BM) and/or peripheral blood (PB) and/or infiltrated extramedullary samples should be done at diagnosis and relapse as critical time-points	91% (32/35)	R1A	5% (2/37)
2.2. Biobanking of BM and/or PB cells should be done at the end of induction and at the end of consolidation	81% (26/32)	R1A	14% (5/37)
2.3. There are no other desirable time-points with the current trial strategy	71% (15/21)	R2	34% (11/32)
2.4. Before storage, blast cells should be concentrated using density gradient centrifugation (e.g., Ficoll, Lymphoprep)	85% (29/34)	R1A	8% (3/37)
2.5. If visible erythrocyte contamination persists in the cell pellet after density gradient centrifugation and separation, red cell lysis should be performed	No consensus	R1A and R2	28% (9/32)
2.6. All remaining cells not used for diagnostics should be biobanked	83% (30/36)	R1A	3% (1/37)
2.7. Viable cells should be stored using 5–50 million cells per vial	72% (23/32)	R1A	14% (5/37)
2.8. Viable cells should be stored in liquid nitrogen	87% (32/37)	R1A	0/37
2.9. If there are less than 5 million cells after density gradient centrifugation and separation, this material should be stored as viable cells (DMSO), non-viable cells (dry pellet), DNA (direct extraction), or other	No consensus	R2	22% (7/32)
2.10. Dry pellets should be stored at −80 °C	87% (27/31)	R1A	16% (6/37)
2.11. The biobank should have information about DNA and RNA isolation and availability (even if the isolations are done in a Diagnostic Lab)	81% (29/36)	R1A	3% (1/37)
2.12. If RNA isolation is not performed by the biobank, storage of viable cells should be prioritized	77% (20/26)	R2	19% (6/32)
2.13. If DNA isolation is not performed by the biobank, storage of viable cells should be prioritized	73% (19/26)	R2	19% (6/32)
2.14. Leukaemic cell percentage should be determined by flow cytometry or morphology after cell isolation	79% (26/33)	R1A	11% (4/37)
2.15. Cell viability should be determined after cell isolation (by flow cytometry or morphology or trypan blue)	91% (19/22)	R2	31% (10/32)
2.16. The following procedural information for obtaining cells should be noted: Red cell lysis required, Cell viability, Time of cryopreservation, Type of cryopreservation solution, Date of cryopreservation solution preparation, Date/time of storage, Number of cells per tube, Blast percentage per tube	74% (26/35)	R1A	5% (2/37)
2.17. A protocol for cryopreservation based on general consensus is recommended to harmonize cryopreservation procedures	93% (31/33)	R1A	11% (4/37)
3. Biobanking of plasma			
3.1. Patients with Acute Lymphoblastic Leukaemia should have plasma biobanked	68% (21/31)	R1B	9% (3/34)
3.2. Plasma should be stored at diagnosis and relapse (bone marrow (BM) and/or peripheral blood (PB)	82% (23/28)	R1B	7% (2/30)
3.3. If BM and PB have been sent to the biobank, the plasma of both specimens should be stored	77% (20/26)	R1B	13% (4/30)
3.4. BM and/or PB plasma should be stored at the end of induction (TP1)	76% (19/25)	R1B	17% (5/30)
3.5. Plasma should be also stored at the end of consolidation (TP2)	83% (10/12)	R2	63% (20/32)
3.6. Plasma storage at other time-point is not required with the current trial strategy (other than diagnosis/Relapse/TP1/TP2)	77% (10/13)	R2	59% (19/32)
3.7. The type of collected material should be recorded by the biobank (BM/PB/etc.)	96% (27/28)	R1B	7% (2/30)
3.8. The total plasma volume per specimen should be stored in the biobank (no limitation of the number of aliquots)	82% (22/27)	R1B	10% (3/30)
3.9. A minimum of 2 aliquots should be stored (0.5–2 mL)	95% (20/21)	R2	34% (11/32)
3.10. If the total plasma volume of the received sample is less than 0.5mL, the material should be stored	86% (19/22)	R2	31% (10/32)
3.11. −80 °C is the preferable method to store plasma aliquots	96% (23/24)	R1B	20% (6/30)
4. Biobanking of serum			
4.1. Patients with Acute Lymphoblastic Leukaemia should have serum biobanked	78% (21/27)	R1B	21% (7/34)
4.2. Serum should be stored at diagnosis and relapse? (bone marrow (BM) and/or peripheral blood (PB)	83% (20/24)	R1B	20% (6/30)
4.3. If BM and PB are received, serum of both specimens should be stored	74% (17/23)	R1B	23% (7/30)
4.4. BM and/or PB serum should be stored at the end of induction (TP1)	82% (18/22)	R1B	27% (8/30)
4.5. BM and/or PB serum should be stored at the end of consolidation (TP2)	67% (14/21)	R1B	30% (9/30)
4.6. Serum storage at other time-point is not required with the current trial strategy (other than diagnosis/Relapse/TP1/TP2)	67% (8/12)	R2	63% (20/32)
4.7. The type of collected material should be recorded by the biobank (BM/PB/etc.)	96% (25/26)	R1B	13% (4/30)
4.8. The total serum volume per specimen should be stored in the biobank (no limitation of the number of aliquots)	78% (18/23)	R1B	23% (7/30)
4.9. A minimum of 2 aliquots should be stored (0.5–2 mL)	95% (18/19)	R2	41% (13/32)
4.10. If the total serum volume of the received sample is less than 0.5mL, the material should be stored	94% (16/17)	R2	47% (15/32)
4.11. −80 °C is the preferable method to store serum aliquots	90% (18/20)	R1B	33% (10/30)
5. Biobanking of Cerebro-Spinal Fluid (CSF)			
5.1. Patients with Acute Lymphoblastic Leukaemia should have CSF specimens biobanked	82% (23/28)	R1B	18% (6/34)
5.2. Biobanking of CSF should be done for ALL patients at diagnosis and at relapse	100% (24/24)	R1B	17% (5/29)
5.3. Biobanking of CSF should be done at follow-up time-points (regardless of infiltration at diagnosis)	81% (13/16)	R1B	45% (13/29)
5.4. CSF specimens should be collected in a dry sterile tube	72% (13/18)	R1B	38% (11/29)
5.5. CSF should be centrifuged for separate storage of supernatant and pellet	83% (15/18)	R1B	38% (11/29)
5.6. Both supernatant and pellet should be stored after sample centrifugation	73% (16/22)	R2	31% (10/32)
5.7. The total volume of CSF specimens should be stored in the Biobank (no limitation of the number of aliquots)	83% (19/23)	R1B	21% (6/29)
5.8. The supernatant should be stored in 0.5mL aliquots	75% (12/16)	R2	50% (16/32)
5.9. If the total CSF volume of the received sample is less than 0.5mL, the material should be stored	No consensus	R2	34% (11/32)
5.10. The preferred storage method for CSF supernatant vials is: Liquid Nitrogen, −80 °C or −20 °C	100% (21/21)	R2	31% (10/32)
6. Biobanking of germline material			
6.1. Patients with Acute Lymphoblastic Leukaemia should have germline material biobanked	94% (29/31)	R1B	9% (3/34)
6.2. Skin biopsy is the preferred option to obtain germline material	77% (17/22)	R1B	31% (10/32)
6.3. If a skin biopsy is not possible, sample at remission (PB or BM with <1% blasts) is acceptable for germline material	83% (20/24)	R1B	25% (8/32)
6.4. A buccal swab is only acceptable for germline material if a skin biopsy or a PB or BM sample at remission (<1% blasts) are not possible	73% (16/22)	R1B	31% (10/32)
6.5. The preferred source of germline DNA material is: skin biopsy without fibroblast culture (direct extraction) or fibroblast culture from skin biopsy	No consensus	R2	25% (8/32)
6.6. For transplanted patients, the DNA of the stem cell donor should be retrieved and stored in the biobank	68% (15/22)	R1B	31% (10/32)
7. Quality monitoring and data			
7.1. Biobanks should be accredited (e.g: ISO 20387:2018)	73% (22/30)	R1B	12% (4/34)
7.2. Biobanks should have an internal quality monitoring of biobanked material	91% (30/33)	R1B	3% (1/34)
7.3. Biobanks should ask users for feedback on the quality of received biobank material	97% (31/32)	R1B	6% (2/34)
7.4. Biobanks should have a standard form for collecting user feedback	94% (30/32)	R1B	6% (2/34)
7.5. Biobanks should collect data on research performed using the released samples (assays performed, e.g. RNAsequencing)	84% (27/32)	R1B	6% (2/34)

### Procedure and collection

Ensuring a high quality of stored samples for research starts with obtaining high-quality fresh samples. Participants strongly agreed on the importance of avoiding haemodilution when large BM sample volumes were required, consensus was reached on reinserting the needle at a different angle through the original puncture site.

No agreement was reached on the preferred type of sample collection tubes, with most votes going to Sodium Heparin and Ethylenediaminetetraacetic acid (EDTA). This in part reflects BM aspiration procedure detailed in the trial protocol (Supplementary Method 1), in which different tubes were deemed acceptable, depending on local or national practices. Also in Round 2, where the question was re-formulated to ask about the best type of tube for processing viable-frozen cells for functional studies, no consensus was reached. Therefore, we conclude that different types of anti-coagulant tubes (Sodium Heparin, EDTA or acid citrate dextrose (ACD)) are used to obtain viable frozen cells, depending on the preferences and processing procedures of the local or national biobanks. For instance, heparin has been found to interfere with nucleic acid amplification assays [5], which is mitigated through density gradient centrifugation and subsequent cell washing steps. If a research study involves the production of immortalised cell lines derived from PB lymphocytes, it is advised to prioritise ACD anti-coagulation [6]. Despite EDTA posing challenges for cytogenetic analysis, it can provide blood fractions suitable for a wide range of DNA-based and protein assays [7]. Importantly, the panel agreed that the type of collection tube should be noted, as well as the time between sampling and processing to allow researchers to evaluate whether the biobanked material is suitable for their research application [8].

### Biobanking of cells

The panel was asked about desired time-points to collect BM/PB cells, methods for cell processing and storage conditions. The panel agreed that cells should be collected at diagnosis, relapse, end of induction and end of consolidation. Additionally, the panel agreed of storing infiltrated extramedullary samples, such as pleural and pericardial fluids, at diagnosis and relapse. Experts did not feel that other time-points were useful for routine biobanking. If RNA and/or DNA were not extracted by the biobank, participants agreed on prioritizing the storage of viable cells rather than keeping cell pellets for future extraction. The panel agreed that all cells not used for diagnostic purposes should be biobanked. Furthermore, if fewer than 5 million cells are recovered after density gradient centrifugation and separation, participants also supported the storage as viable cells, since this leaves all options open for functional studies such as drug sensitivity testing and patient-derived xenografts. This is valuable information, leading to the strong recommendation that biobanks should prioritize storage of viable cells. On the number of cells per vial to store, participants frequently suggested not to exceed 20M cells, both for optimal recovery and to allow multiple applications. Example algorithms how to balance number of vials and number of cells per vial are provided (Supplementary Method 2).

Concerning processing of BM aspirate or PB samples to obtain cells, there was agreement on the use of density gradient centrifugation. The attitude towards dealing with visible erythrocyte contamination in the cell pellet after density gradient centrifugation and separation did not reach consensus. Some participants considered that minimising the number of protocol steps for not losing cells is more important than purity or that visible contamination by erythrocytes is too suggestive. This is in sharp contrast to others for whom this step is part of their routine practice to avoid interference of haemoglobin in colorimetric assays including MTT and debris in flow cytometry measurements. Moreover, DNA and RNA from (immature) red blood cells and DNA sticking to the surface of mature red blood cells interferes with DNA and RNA quantification [9, 10]. After density gradient centrifugation, the panel agreed that cell viability should be assessed but did not reach consensus on a single preferred technique. Flow cytometry and trypan blue are equally accepted. There was strong agreement on implementing a consensus-based protocol to standardise cryopreservation procedures. The ALLTogether1 laboratory manual contains a standard operation procedure for cryopreservation of viable cells needed for an ALLTogether1 protocol-related sub-study (Supplementary Method 3). This procedure was optimized for recovery of viable lymphoblasts after thawing, with the most critical step that the cell suspension and freezing medium need to be cold (ice water, 0–4 °C) and cryovials pre-cooled in −20 °C freezer to minimize the toxic effect of DMSO. Laboratories who implemented this procedure reported improved viability upon thawing from typically <30% to >80% (personal communication to Judith Boer from Máxima Biobank and LBL2018 protocol Biobank).

### Biobanking of plasma and serum

Currently, plasma and serum from BM/PB samples are stored in the majority of ALLTogether study group biobanks (Supplementary Table 1) and could be useful for the study of cytokines, for example [11]. Experts agreed that plasma and serum should be stored at diagnosis, relapse, end of induction and end of consolidation. Experts did not consider other time-points as critical within the current trial strategy. However, the majority of experts abstained from statements 3.6 and 4.6 (abstention rate of 59% for plasma and 63% for serum) in Table 1, possibly suggesting lack of experience with research on this material. Some experts suggested that additional time-points might be valuable, particularly in context of cell-based immunotherapies to evaluate response. One expert proposed storing plasma monthly after consolidation to enable the discovery of biomarkers predicting relapse.

### Biobanking of cerebrospinal fluid (CSF)

Optimizing treatment of the central nervous system (CNS) remains a challenge in ALL. Research focusing on CNS-ALL is needed to provide a better understanding of disease biology as well as putative drug targets and biomarkers. CSF-Flow, a sub-study within the ALLTogether1 trial, is a study on identifying biomarkers to enable accurate prediction of relapse-risk [12]. Hence, the establishment of a robust CSF biobank is deemed indispensable [13, 14]. Experts reached consensus that CSF should be stored at diagnosis and relapse and at follow-up regardless of infiltration at diagnosis. Day 15 and day 29 time-points are obligatory as part of a sub-study in the current trial. Experts agreed that after centrifugation, the supernatant and pellet should be stored. Storage of the pellet (as non-viable cells) would allow for DNA, RNA, or protein extraction. There was agreement not to biobank CSF at other follow-up time-points. There was no clear consensus on the storage method, however, 86% of answering experts considered −80 °C acceptable, while 55% preferred liquid nitrogen. Multiple experts considered both options, possibly reflecting the dependence on the biological questions posed. Supplementary Table 2 summarise CSF storage practices across the consortium.

### Biobanking of germline material

Whole exome sequencing (WES) and whole genome sequencing (WGS) are frequently used in research for the detection of genomic aberrations and are starting to be used as routine diagnostic tools. DNA isolated from germline material is critical for discrimination between somatic and germline variants. Gathering information across the consortium showed that one country has successfully implemented skin biopsies along WES/WGS into routine workflows, minimizing patient burden while ensuring timely results. The panel agreed on the necessity to store germline material, with the preferred source being a skin biopsy (77% agreement). Remission samples (<1% leukaemic cells) were also deemed acceptable, while buccal swabs were considered the least preferred option. The writing group suggested that direct DNA extraction from skin biopsy without fibroblast culture to avoid culture time would be a preferred option to expedite WGS/WES analysis, notwithstanding the risk of potential low contamination by leucocyte-derived DNA. However, the panel found several sources of germline DNA equally suitable, including direct DNA isolation or fibroblast culture from skin biopsy, remission PB or BM. This likely reflects divergent local practices among the participants, such as WGS/WES not being routine diagnostics or as skin biopsy not being a procedure performed in every centre. Indeed information gathered from national biobanks indicated that this source of material is not routinely stored across the consortium. Skin biopsy with culture fibroblasts is the preferred method for confirmation of germline predisposition to haematological malignancies [15, 16]. Cell culture offers advantages such as increased DNA yield and enables cell storage for potential functional studies but takes a few weeks and can fail in rare cases. For transplanted patients, the panel agreed (68% agreement) that DNA of the stem cell donor should be retrieved and stored in the biobank. Participants highlighted the requirement of donor consent for storage.

### Quality and data monitoring

The experts agreed (73%) on the necessity for biobanks to obtain ISO accreditations to ensure high sample quality. The standard ISO 20387:2018 on “Biotechnology — Biobanking — General requirements for biobanking" [17] provides guidelines and requirements for the establishment, operation, and management of biobanks. This standard outlines procedures related to quality management, sample collection, storage, retrieval, and transportation to ensure the integrity and traceability of biological specimens. ISO/TR 22758:2020 “Biotechnology - Biobanking - Implementation guide for ISO 20387” provides support for implementing the requirements of ISO 20387:2018 [18]. Currently, only one ALLTogether affiliated biobanks has obtained ISO accreditation. The survey did not include questions specifically addressing infection control monitoring, such as Mycoplasma infection, since ISO 20387: 2018 [13] does not require specific protocols. Mycoplasma is a well-known contaminant in established cell culture settings altering the phenotypic and functional characteristics of cells in vitro [19, 20] but has not been described in short-term cultures of primary cells. Within the consortium, biobanks handling primary patient samples do not have Mycoplasma testing protocol in place.

The experts reached strong agreement (91%) on the recommendation for internal quality monitoring of biobanked material. Suggestions to perform this included at least a yearly review of nucleic acid integrity (DIN/RIN values) and cell viability pre-freezing and post-thawing. The panel strongly agreed (97% agreement) that a valuable method to assess the quality of biobanked samples would be to request feedback from researchers receiving cryopreserved samples on the number of recovered cells and the viability percentage after thawing. An example of a yearly annual report form from VIVO Biobank in UK can be found on https://vivobiobank.org/researchers/applying. Another example of satisfactory survey template developed by the Biobanking and BioMolecular Resource Research Infrastructure – European Research Infrastructure Consortium (BBMRI-ERIC) can be found on https://www.bbmri-eric.eu/services/quality-management/.

## Discussion

**Fig. 2:**
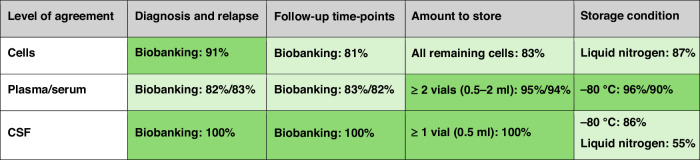
Summary of the Delphi consensus with level of agreement percentage. Green colour intensity is proportional to the level of agreement regarding the biobanking of cells, plasma/serum and CSF.

The establishment of the ALLTogether consortium has created a unique framework for scientific collaboration between study groups across Northern and Western Europe. Successful translation of basic scientific studies into improved patient outcomes relies on access to large and well-annotated cohorts. Establishing best practice for biobanking for ALL is crucial to ensure storage of valuable biospecimen across the ALLTogether consortium. Using a modified Delphi method, we provided a consensus documentation on best biobank practices within the consortium. The surveys resulted in several strong recommendations (summarised in Fig. 2). First, biobanks should allocate effort and resources to store all viable cells from diagnosis, relapse (PB, BM and extra-medullary samples) and follow-up time-points post-induction and post-consolidation, enabling retrospective extraction of DNA, RNA, and (phospho-)proteins if not initially performed. Various types of cell storing scenarios could be considered, balancing the number of vials and the number of cells per vial needed for different applications: small vials (5–10 million cells) for backup diagnostics, RNA/DNA/protein isolation or for generation of patient-derived xenografts (PDX); medium-sized vials (10–20 million cells) for most experiments including single cell sequencing, and larger or multiple vials for functional studies with multiple culture conditions, e.g. drug library screening. Using proven cryopreservation methods, a cell recovery rate between 50% and 80% can be achieved, which contributes to optimal use in research. In addition to the cell storage, the collection and storage of the plasma and serum is highly recommended. A third strong recommendation of the survey is the storage of CSF (supernatant and pellet) at diagnosis, relapse and follow-up time-points day 15 and post-induction. This facilitates biomarker research on CNS-ALL, which is the focus of a sub-study of the ALLTogether1 trial. While this approach preserves an uncontaminated supernatant for future studies, it limits the scope of research requiring viable cells. However, since leukaemic cells often adhere to stroma, studying soluble biomarkers such as leukemic-derived vesicles, secreted proteins and metabolites, or cell-free DNA may prove more relevant for understanding CNS disease characteristics.

Given the current trial strategy, experts considered that additional collection time-points were not deemed essential (statements 2.3 and 3.6 and 4.6 for cells, DNA, RNA, serum, plasma). Without a clearly defined strategy and research plan, experts felt that collecting additional samples, such as after end of treatment, exclusively for biobanking would not be justified, even if they could provide valuable information, for example regarding long-term treatment effects and potential late toxicities.

Other significant recommendations emphasize the importance of biobanks receiving feedback from researchers on sample quality and data generated from these samples. Quality metrics would help the biobanks to potentially review their practices but also inform future researchers using the same samples. Another important role of user feedback includes publications and presentations using biobank resources, as the acknowledgement of the valuable contribution of biobanks helps to ensure their sustainability [21]. In addition, the value of a specimen increases significantly with the amount of information available about it. Our expert panel recommended for biobanks to document the methods applied to biobanked samples, such as sequencing, enabling data sharing with other researchers when original materials are unavailable. This approach enhances transparency and facilitates broader access to valuable research resources and findings. The Biobank Committee's future endeavours also involve incorporating PDX samples generated by ALLTogether research projects. These samples serve as valuable preclinical cancer models, crucial for translational research aimed at validating the therapeutic effectiveness of compounds or exploring novel therapeutic strategies, thus advancing precision medicine initiatives.

Six statements from the survey failed to reach a consensus, despite attempts at reformulation or clarification in the second round. These related to questions about collection tubes, cell processing for cryopreservation, and source of germline DNA. The rate of panel members giving ‘no opinion’ was above 20% for these questions, suggesting the requirement of specific expertise. While our survey explored many aspects of essential materials and time-points for biobanking of ALL, other crucial aspects of biobanking deserve attention such as ethics, legal considerations, operations (small, centralised or multisite biobanks), and financial sustainability. These elements were beyond the scope of this specific investigation and may require a different range of specialties represented on the panel. Ultimately, while the survey aimed to establish harmonized guidelines, we recognize the need for flexibility in their application to ensure feasibility and equity across diverse healthcare systems. In high-resource settings, biobanks may have the infrastructure, funding, and expertise to implement the full suite of recommendations, including advanced protocols such as the collection of skin biopsies for germline material. In contrast, in low-resource settings, logistical and financial barriers may necessitate prioritizing less resource-intensive practices, such as the use of remission samples for germline material when WES/WGS is not yet routinely implemented. Addressing these disparities will require tailored strategies and ongoing collaboration between biobanking networks. Our recommendations could serve as a roadmap for identifying critical gaps in current practices and building a case for additional funding or resources. Engaging stakeholders, including hospital administrations, policymakers, and funding bodies, to highlight the importance of biobanking in advancing research and improving patient outcomes, may further support the implementation of these practices even in challenging settings.

In the context of precision and personalized medicine, it is important for biobanks to transition towards a patient-centred approach [22]. ALLTogether consortium has actively engaged Patient and Public Involvement (PPI) representatives to ensure their meaningful contribution and feedback on various aspects including research projects, sample availability, clinical unmet needs, and ethical and legal considerations surrounding the clinical trial. Several national biobanks within the consortium have Patient Representatives integrated into their committees.

## Conclusion

The comparison of current practices of ALLTogether consortium related biobanks facilitated knowledge sharing and fostered collaboration among biobanks. The Delphi survey across the consortium resulted in recommendations that could serve as guidelines for initiating or updating procedures based on consensus to facilitate collaborative research studies on samples from patients with ALL.

## Supplementary information

Supplemental information

Appendix 1: List of Members of the Biobanking Committee of ALLTogether consortium

Supplemental Table 1: Questions and answers per participants of the three surveys (Excel)

## Data Availability

All data used in this project are available in the Supplementary Data.
