# Supplementary material for: ALLTogether recommendations for biobanking samples from patients with acute lymphoblastic leukaemia: a modified Delphi study
Source: Br J Cancer. 2025 Feb 22;132(6):493–501. doi: 10.1038/s41416-025-02958-x (PMC11920285; doi:10.1038/s41416-025-02958-x)
Supplement: Supplementary file 1 — Supplemental information [file 41416_2025_2958_MOESM1_ESM.docx]

**Supplemental information for**

**ALLTogether recommendations for biobanking samples from patients with acute lymphoblastic leukaemia: a modified Delphi study**

Amélie Trinquand et al.

**SUPPLEMENTAL METHODS**

Supplemental Methods 1: Bone marrow aspiration procedure ALLTogether1

Supplemental Methods 2: Examples of viable cell banking algorithm

Supplemental Methods 3: ALLTogether1 investigator laboratory manual standard operating procedure for cryopreservation of viable cells

**SUPPLEMENTAL FIGURE**

Supplemental Figure: Geographical spread of responders per round. Countries are presented by alphabetical order.

**SUPPLEMENTAL TABLES**

Supplemental Table 1: Questions and answers per participants of the three surveys (Excel)

Supplemental Table 2: Biobanking infrastructures and material stored across the ALLTogether consortium

**SUPPLEMENTAL METHODS**

**Supplemental Methods 1: Bone Marrow aspiration procedure ALLTogether1**

The quantity, quality and representative nature of samples for disease response assessment in the ALLTogether trial is critical. It is therefore essential to follow these guidelines when taking samples.

**Consider using aspirate needles coated with heparin before aspiration (to avoid clot)**

**At diagnosis**

*Aspirate 1 - in order*

1. 0.25 ml direct spread morphology

2. 1 ml EDTA diagnostic flow cytometry*

3. 0.5 ml cell culture medium – diagnostic genetics^

4. 2 ml EDTA – molecular genetics

*Aspirate 2 – re-insert aspirate needle at different angle through same skin puncture; in order*

1. 5 ml into single syringe, mix gently, divide equally between flow MRD and PCR MRD in EDTA or Na-, Li- Heparin**

2. **5 ml biobanking (depending on local arrangements)**

3. Local research samples

**At follow-up timepoints**

*Aspirate 1 - 5ml max (if more there is a risk for increased blood contamination)*

1. 5 ml into single syringe, mix gently, divide between flow MRD (3ml) and PCR MRD

(2ml) in EDTA or Na-, Li- Heparin**

*Aspirate 2 – re-insert aspirate needle at different angle through same skin puncture; in order*

1. 0.5ml morphology

2. **5ml biobanking (depending on local arrangements)**

3. Local research samples

**Notes**

*diagnostic and MRD flow labs may not be co-located so a separate sample required. Can be omitted if diagnostic and MRD flow labs are the same laboratory.

^ If required – check local genetics requirements

** Na-, Li- Heparin or EDTA acceptable – liaise with local MRD labs for requirements

**Protocol for inaspirable patients**

**Principles: Diagnosis, flow cytometry, flow and molecular MRD and genetic analysis can all be performed from cells or DNA extracted from trephine samples**

*Aspirate 1*

If any volume at all can be aspirated, make 2 slides, residual in EDTA/Li Heparin for diagnostic flow.

*Trephine 1*

Make trephine roll, send trephine in formalin for histopathology (especially if completely inaspirable)

*Trephine 2*

In saline for flow MRD

*Trephine 3*

In saline for DNA extraction for MRD

*Trephine 4*

In saline for DNA extraction for genetics (only if genetics not co-located with MRD)

**Supplemental Methods 2: Examples of viable cell banking algorithm**

**VIVO biobank, UK**

• Up to 20x10^6^ cells – equal aliquots of ~ 5x10^6^ cells

• 20 - 40x10^6^ cells 4 aliquots at 5x10^6^ cells

• Up to 60x10^6^ cells 6 aliquots at 5x10^6^ cells, 1 aliquot at 10x10^6^ cells

• Up to 70x10^6^ cells 6 aliquots at 5x10^6^ cells, 2 aliquot at 10x10^6^ cells

• > 70x10^6^ cells 6 aliquots at 5x10^6^ cells, increasing numbers of aliquots of 10x10^6^ cells up to 150x10^6^ cells when additional aliquots of 50x10^6^cells

**Lisbon biobank, Portugal**

• Up to 60x10^6^ cells – 1 to 6 equal aliquots 5-10x10^6^ cells

• Up to 140x10^6^ cells – 6 aliquots at 10x10^6^ cells, 1-4 equal aliquots 10-20x10^6^ cells

• Up to 200x10^6^ cells – 6 aliquots at 10x10^6^ cells, 4 equal aliquots 20x10^6^ cells, 1-2 aliquots 20-30x10^6^ cells

• Up to 300x10^6^ cells – 6 aliquots at 10x10^6^ cells, 4 equal aliquots 20x10^6^ cells, 2 aliquots 30x10^6^ cells, 2 vials 30-50x10^6^ cells (increase number of aliquots at 50x10^6^ cells if needed).

**PHO Biobank Ghent (BSPHO), Belgium**

• Up to 10x10^6^ cells – equal aliquots of ~ 5x10^6^ cells

• 10 - 200x10^6^ cells - 3 equal aliquots at ~5-10x10^6^ cells, aliquots at ~ 50x10^6^ cells

• > 200x10^6^ cells - 3 equal aliquots at ~5-10x10^6^ cells, aliquots at ~ 50x10^6^ cells up to 200 x10^6^ cells, remainder at max 200x10^6^ cells

**Uppsala BioLab (Uppsala Biobank, Region Uppsala which is the host of NOPHO Leukemia Biobank), Sweden**

• Up to 10x10^6^ cells – equal aliquots of ~ 5x10^6^ cells

• 10x10^6^ -100x10^6^ cells – 2-3 aliquots at 10*10^6^ if material is still left at ~ 20*10^6^

• 100 - 200x10^6^ cells –aliquots at ~ 30x10^6^ cells

• > 200x10^6^ cells - aliquots at ~ 30x10^6^ cells up to 300 x10^6^

**Supplemental Methods 3: ALLTogether1 investigator laboratory manual standard operating procedure for cryopreservation of viable cells**

*Materials:*

- 2 ml cryopreservation vials
- Culture medium containing at least 20% fetal calf serum (FCS) or 100% FCS
- 100% DMSO
- Ice (no dry ice, but regular ice)
- Styrofoam box

*Method: (for one vial)*

1. Dissolve the required amount of leukemic cells in 1 ml of culture medium or 100% FCS.
2. Make cryopreservation medium containing 20% DMSO:
   1. 800 μl culture medium + 200 μl 100% DMSO
   2. 800 μl 100% FCS + 200 μl 100% DMSO

Of note: add DMSO dropwise while shaking gently to avoid precipitation of serum proteins.

1. Pre-cool the cells and the cryopreservation medium on ice (at least 10 minutes).

If both suspensions have cooled down, add 1 ml of cryopreservation medium to 1 ml of the cell suspension dropwise while shaking gently. Final concentration of DMSO needs to be 10%.

1. Quickly transfer 2 ml of the cell-cryosuspension to a cryopreservation vial (if many vials are processed simultaneously, pre-cool vials at -20°C for 10 minutes; always keep solutions and vials on ice till transfer to styrofoam box/-80°C).
2. Transfer the vial(s) a.s.a.p. to a styrofoam box (having a thin wall of ~1 cm) and store at -80°C for at least 24hrs to a maximum of 72 hrs.
3. Store vial(s) in a liquid nitrogen tank.

**SUPPLEMENTAL FIGURE**

Supplemental Figure: Geographical spread of responders per round. Countries are presented by alphabetical order.

**
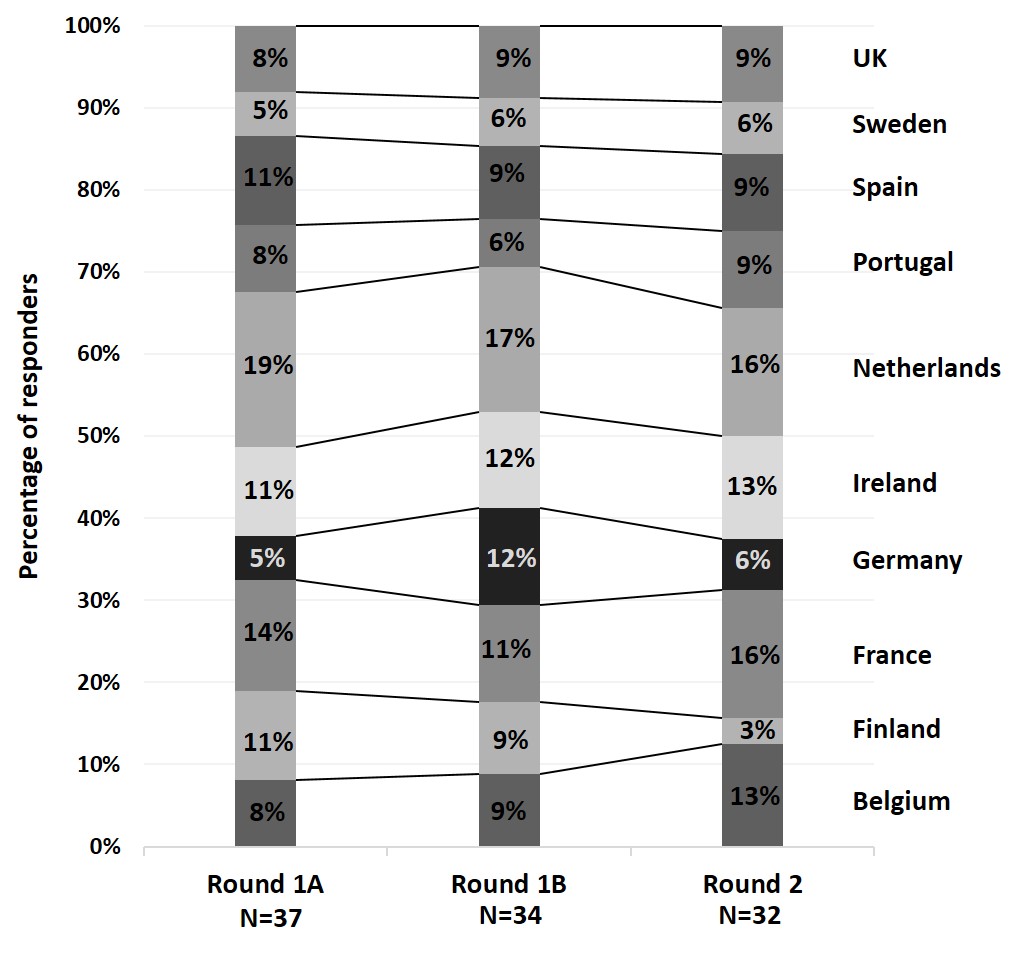
**

**SUPPLEMENTAL TABLES**

**Supplemental Table 1: Questions and answers per participants of the three surveys**

See separate Excel table.

**Supplemental Table 2: Biobanking infrastructures and material stored across the ALLTogether consortium**

| **Countries**  **(study groups/ biobank)** | **Site** | **Attached to diagnostic laboratory** | **Viable cells** | **Cell pellet** | **DNA** | **RNA** | **Plasma/ serum** | **CSF** | **Skin biopsy** | **Collection tube: viable cells** | **Collection tube: plasma** | **Collection tube: serum** |
| --- | --- | --- | --- | --- | --- | --- | --- | --- | --- | --- | --- | --- |
| Belgium (BSPHO) | Central | Yes | Yes | Yes | Yes | Yes | Yes | Yes | No | EDTA/ Sodium Heparin | Sodium Heparin | Clot activator |
| Finland  (FHRB) | Central | No | Yes | Yes | Yes | Yes | Yes | No | Yes | EDTA | EDTA and lithium heparin | All serum tubes accepted |
| France  (SFCE) | Multiple  (2sites) | Yes | Yes | Yes | Yes | Yes | Yes/No* | Yes† | No* | EDTA/ Sodium Heparin | Sodium Heparin |  |
| Germany (CoALL) | Central | Yes | Yes | Yes | Yes | No* | No* | Yes† | No | EDTA/ Sodium Heparin |  |  |
| Ireland  (PHOAI) | Central (UK) | No | Yes | Yes | No | No | Yes | Yes† | No | ACD | EDTA | Not collected^§^ |
| Portugal  (GPLP-SHOP) | Multiple | Yes | Yes | Yes | No | No | Yes | Yes† | No | Sodium Heparin | Sodium Heparin | Clot activator |
| Spain  (SEHOP) | Multiple | Yes | Yes | No | No* | No* | Yes | Yes† | No | EDTA | EDTA | Clot activator |
| Sweden (NOPHO) | Central | No | Yes | Yes | Yes | Yes | Yes | Yes† | No | Sodium Heparin | EDTA | Clot activator |
| Netherlands (Máxima) | Central | Yes | Yes | Yes | Yes | Yes | Yes | Yes | Yes | Sodium Heparin | Sodium Heparin | Clot activator |
| United Kingdom (UKALL) | Central | No | Yes | Yes | No | No | Yes | Yes† | No | ACD | EDTA | Not collected^§^ |

* Not routinely; ^§^plan to collect in the future; †supernatant and pellet (non-viable cells)
