# Supplementary material for: ALLTogether recommendations for biobanking samples from patients with acute lymphoblastic leukaemia: a modified Delphi study
Source: Br J Cancer. 2025 Feb 22;132(6):493–501. doi: 10.1038/s41416-025-02958-x (PMC11920285; doi:10.1038/s41416-025-02958-x)
Supplement: Supplementary file 2 — Appendix 1: List of Members of the Biobanking Committee of ALLTogether consortium [file 41416_2025_2958_MOESM2_ESM.docx]

**Appendix 1: List of Members of the Biobanking Committee of ALLTogether consortium.**

Listed per study group:

| **Study Group** | **Members** | **Affiliations** |
| --- | --- | --- |
| PMC  Princess Máxima Center for Pediatric Oncology | Judith Boer  Valérie de Haas  Marion Persoon | Princess Máxima Center for Pediatric Oncology, Utrecht, The Netherlands |
| UKALL  The United Kingdom ALL Group | Anthony Moorman | Leukaemia Research Cytogenetics Group, Centre for Cancer, Translational and Clinical Research Institute, Newcastle University, United Kingdom |
|  | Anne Thomson | VIVO Biobank, Milton Keynes, United Kingdom |
| PHOAI  The Paediatric Haematology/Oncology Association of Ireland | Jonathan Bond | Systems Biology Ireland, School of Medicine, University College Dublin, Dublin, Ireland  National Children’s Cancer Service, Children’s Health Ireland at Crumlin, Dublin, Ireland |
|  | Amélie Trinquand | National Children’s Cancer Service, Children’s Health Ireland at Crumlin, Dublin, Ireland |
| NOPHO  Nordic Society of Paediatric Haematology and Oncology | Karolin Bergenstråhle | Uppsala Biobank,Uppsala University, Uppsala, Sweden |
|  | Tiina Vesterinen | Institute for Molecular Medicine Finland (FIMM), HiLIFE, University of Helsinki, Helsinki, Finland |
|  | Maria Thastrup | Department of Pediatrics and Adolescent Medicine, Rigshospitalet, University of Copenhagen, Copenhagen, Denmark |
| COALL  Co-operative study group for childhood acute lymphoblastic leukemia | Udo zur Stadt  Lena Behrmann | Paediatric Haematology and Oncology, University Medical Centre Hamburg-Eppendorf, Hamburg, Germany |
| BSPHO  Belgian Society of Paediatric Haematology Oncology | Tim Lammens | Department of Internal Medicine and Pediatrics, Ghent University, Ghent, Belgium  Department of Pediatric Hematology-Oncology and Stem Cell Transplantation, Ghent University Hospital, Ghent, Belgium  Cancer Research Institute Ghent, Ghent, Belgium |
|  | Anne Uyttebroeck | Department of Paediatric Haematology and Oncology, University Hospitals Leuven, Leuven, Belgium |
| SFCE  Société Française de lutte contre les Cancers et leucémies de l’Enfant et de l’adolescent | Aurélie Caye | Service de de Génétique Moléculaire, Hôpital Robert Debré, GHU AP-HP Nord - Université Paris Cité, Paris  APHP-Centre de ressources biologiques (CRB), Hôpital Robert Debré, Paris, France |
|  | Marion Strullu | Service d'Hémato-Immunologie pédiatrique, Hôpital Robert Debré, GHU AP-HP Nord - Université Paris Cité, Paris, France |
| SEHOP  Sociedad Española de Hematología y Oncología Pediátricas | Cristina Jou Munoz | Hospital Niño Jesús, Madrid, Spain |
| GPLP-SHOP  Grupo Português de Leucemias Pediatricas member of the Sociedade de Hematologica e Oncologia Pediatrica | Ana Lúcia Barbosa  Paula Gameiro | Department of Haematology, Instituto Português de Oncologia, Lisbon, Portugal |
| AYA representative (Adolescent and Young Adult) | Anna Castleton | Haematology Department, The Christie Hospital NHS Foundation Trust, Manchester, United Kingdom |
